# Metabolic-associated steatotic liver disease in children and adolescents: a scoping review and narrative synthesis of epidemiology, risk factors, and screening approaches with emerging implications for sub-Saharan Africa

**DOI:** 10.3389/fendo.2026.1784970

**Published:** 2026-03-13

**Authors:** Bruno Basil, Peace Ngozi Okoro

**Affiliations:** 1 International Institute of Pathology and Forensic Science Research, David Umahi Federal University of Health Sciences, Uburu, Nigeria; 2 Department of Medicine, David Umahi Federal University of Health Sciences, Uburu, Nigeria

**Keywords:** African paradox, double burden of malnutrition, epidemiology, paediatric MASLD, sub-Saharan Africa, waist-to-height ratio

## Abstract

**Background:**

Metabolic-Associated Steatotic Liver Disease (MASLD) is recognized as one of the most common chronic liver diseases in children globally, rising in tandem with the childhood obesity pandemic. Although high-income countries focus on advanced phenotyping, Sub-Saharan Africa (SSA) faces a distinct “two-speed” epidemic characterized by rapid urbanization and a unique “double burden” of malnutrition and obesity. This review examines the global and regional epidemiology of paediatric MASLD, contrasting established Western practices with the unique genetic, environmental, and diagnostic challenges of SSA.

**Methods:**

A scoping review was conducted following the PRISMA-ScR guidelines and using the JBI methodological framework. PubMed/MEDLINE, Embase, and African Journals Online (AJOL) were searched for literature published between 2010 and 2025 focusing on epidemiology, risk factors, and diagnostic performance in children aged 0–19 years. Evidence was synthesized to compare global prevalence patterns with emerging African data and to evaluate the validity of conventional screening approaches in resource-limited settings.

**Results:**

A total of 68 studies were included. Global evidence estimates paediatric MASLD prevalence between 7.6% and 14% in the general population and as high as 41% among children with obesity. In SSA, data remain sparse but alarming, with pooled prevalence among overweight children reaching 31.1%, a figure derived mostly from studies in the NAFLD-era utilizing ultrasound or ALT proxies, which may not align perfectly with newer MASLD criteria. The region exhibits a distinct “African Paradox” with a lower frequency of the *PNPLA3* genetic risk variant (13.7%), which contributes to lower hepatic steatosis on imaging despite pronounced insulin resistance. As a result, reliance on alanine aminotransferase (ALT) and ultrasonography for screening risks under-detection, obscuring the metabolically high-risk yet hepatically lean phenotype common in SSA. Furthermore, environmental drivers such as high-fructose diets and endocrine-disrupting chemicals may be overriding genetic protection.

**Conclusion:**

Paediatric MASLD in SSA reflects a multifactorial pathology likely driven by environmental stressors, epigenetic “thrifty phenotype” programming, and rapid nutritional transition rather than simple caloric excess. Western-calibrated diagnostic algorithms are poorly suited to the African metabolic phenotype. To mitigate a future surge in advanced liver disease, public health strategy must prioritize low-cost innovations, including validating scalable markers such as Waist-to-Height Ratio (WHtR) and integrating task-shifting approaches within existing HIV and diabetes care platforms.

## Graphical Abstract

**Figure:**
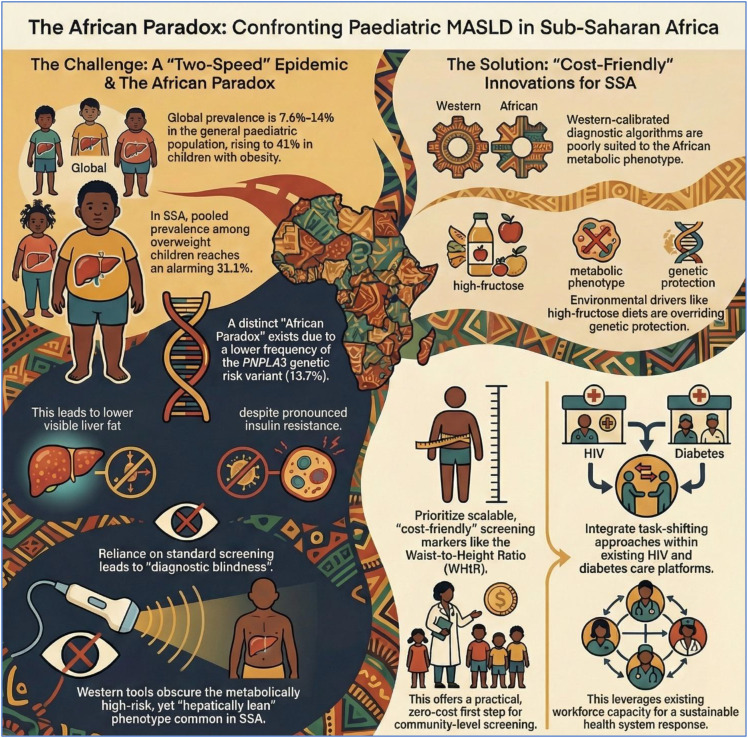

## 1 Introduction

### 1.1 Background

The epidemiology of paediatric diseases has experienced a profound transformation in the first quarter of the 21st century. As global progress in infectious disease control continues to reduce childhood mortality, non-communicable diseases (NCDs) have emerged as a dominant threat to long-term child and adolescent health. Central to this transition is the unprecedented rise in childhood obesity, a trend that has triggered the rapid emergence and recognition of Metabolic-Associated Steatotic Liver Disease (MASLD) as a major paediatric condition (1–3). Formerly known as Non-Alcoholic Fatty Liver Disease (NAFLD), MASLD has now become known as one of the most common chronic liver diseases in children and adolescents worldwide (4). It is characterized by hepatic fat accumulation in the absence of significant alcohol intake and is closely linked to metabolic dysfunction, including obesity, insulin resistance, and dyslipidaemia (4, 5) Global prevalence estimates range from 7.6% to 10% in general paediatric populations and climb to 34–41% among children with obesity, although some contemporary multinational estimates report prevalence figures of 13–26%, reflecting both heterogeneity in methodologies and rising trends over time (1, 6, 7). Once considered a benign adult-onset disorder, MASLD is now seen in children as young as toddlers, representing a substantial shift in the age distribution and natural history of the disease (8, 9).

The last two and a half decades marks a critical era in understanding paediatric MASLD. During this period, researchers have increasingly distinguished a “paediatric phenotype” of fatty liver disease histologically characterized by periportal rather than perisinusoidal fibrosis, and by metabolic profiles distinct from those observed in adults (9, 10). The risk profile has expanded beyond caloric excess to encompass high dietary fructose intake, genetic susceptibility (including variants such as PNPLA3), perinatal programming (maternal obesity, gestational diabetes), and environmental exposures, especially Endocrine-Disrupting Chemicals (EDCs) (11–14).

Globally, MASLD remains largely asymptomatic in its early stages, yet it poses a significant risk for progression to steatohepatitis, fibrosis, cirrhosis, hepatocellular carcinoma, and extrahepatic complications such as type 2 diabetes mellitus (T2DM) and cardiovascular disease (CVD) (15–20). In Sub-Saharan Africa (SSA), the epidemiology of paediatric MASLD remains incompletely defined, but emerging evidence suggests a rising burden associated with rapid urbanization, lifestyle transitions, and increasing rates of obesity and metabolic syndrome (7, 21). The region faces a unique “double burden” of malnutrition, wherein early-life undernutrition (e.g., stunting) followed by exposure to an obesogenic environment confers heightened vulnerability to metabolic dysfunction and hepatic steatosis (22–24). Also compounding these challenges is the “African Paradox,” a metabolic phenotype in which populations of African ancestry often exhibit lower hepatic fat content on imaging despite high insulin resistance, a pattern likely influenced by genetic variants including PNPLA3 (25–27). This phenomenon risks misclassification when screening algorithms developed in non-African populations are applied without local adaptation.

### 1.2 Rationale for the review

The rationale for this scoping review stems from the observation of a “two-speed epidemic” of paediatric MASLD across global regions. In High-Income Countries (HICs), childhood obesity rates, though high, are stabilizing and research efforts have shifted toward mechanistic insights, advanced phenotyping, development of polygenic risk scores, and emerging therapeutic trials (28, 29). In Low- and Middle-Income Countries (LMICs), particularly SSA, childhood obesity is rising rapidly, outpacing the capacity of health systems to respond. Health infrastructure, already burdened by infectious diseases and undernutrition, is ill-equipped to address the emerging metabolic epidemic (30, 31).

Despite the growing importance of paediatric MASLD, SSA remains significantly underrepresented in the global guidelines, which are largely derived from Caucasian, Hispanic, Middle Eastern, and East Asian populations. Screening recommendations for children and adolescents vary widely in terms of age of initiation, BMI thresholds, and diagnostic approaches (32, 33). These inconsistencies are amplified in resource-constrained settings, where advanced imaging modalities (e.g., MRI-PDFF, elastography) are limited and screening is often opportunistic rather than systematic (34–36). Additionally, emerging risk modifiers unique or highly relevant to SSA including perinatal undernutrition, environmental toxins, genetic variation, and potential interactions with antiretroviral therapy (ART) exposure, remain insufficiently examined in current global literature. Understanding these factors is essential for developing locally relevant screening strategies appropriate for the SSA.

### 1.3 Study aim

This scoping review aims to comprehensively map the global and regional research findings on paediatric MASLD between 2010 and 2025, with a special emphasis on Sub-Saharan Africa. It moves beyond simple prevalence summaries to interrogate the heterogeneity of the disease across populations, resource contexts, and environmental exposures.

The specific objectives are to:

- To summarize MASLD prevalence across West, East, Central, and Southern Africa, including data from high-risk paediatric clinics.
- To identify core MASLD risk factors and distinguish them from region-specific modifiers such as genetics, perinatal factors, environmental exposures, and ART.
- To assess how well ALT, AST, and ultrasound perform as screening tools where advanced imaging is unavailable.
- To outline policy and clinical implications, emphasizing the need for locally adapted diagnostic algorithms and strengthened health-system capacity in SSA.

## 2 Methods

### 2.1 Methodological design

This study employed a hybrid Scoping-Narrative Review design. This approach was selected to rigorously map the available evidence on paediatric MASLD epidemiology and risk factors (scoping component) while allowing for a critical, thematic synthesis of complex mechanisms such as the “African Paradox” and diagnostic challenges in resource-limited settings (narrative component). The review was guided by the methodological framework established by Arksey and O’Malley and further refined by the Joanna Briggs Institute (JBI) Manual for Evidence Synthesis (2024) (37). This framework ensures a transparent and replicable search process while providing the flexibility to incorporate diverse evidence sources, including grey literature and expert consensus guidelines relevant to Sub-Saharan Africa.

### 2.2 Eligibility criteria

Eligibility criteria were defined using the Population, Concept, and Context (PCC) framework (37).

- *Population*: Children and adolescents aged 0 to 19 years. Studies with mixed-age cohorts were included only if age-stratified data for the paediatric subgroup were available.
- *Concept*: Steatotic liver disease associated with metabolic dysfunction. To capture the evolution of terminology, studies using NAFLD, MAFLD (2020 definition), and MASLD (2023 Delphi consensus) were all included (4, 5, 8). Studies focusing solely on alcohol-associated liver disease, viral hepatitis, or autoimmune liver disease without metabolic co-factors were excluded.
- *Context*: The review considers global evidence but prioritizes studies from Sub-Saharan Africa (SSA) and other resource-limited settings. To capture the evolution of terminology, studies using NAFLD, MAFLD (2020 definition), and MASLD (2023 Delphi consensus) were all included. It is important to note that the majority of included epidemiological data are derived from studies utilizing NAFLD or MAFLD criteria, which may influence comparability with newer MASLD-specific cohorts.
- *Types of Sources*: Peer-reviewed original research (observational, interventional), systematic reviews, meta-analyses, and clinical guidelines published between January 2010, and May 2025 were included. Case reports were excluded unless they described unique phenotypes relevant to the African context (e.g., steatosis with malnutrition).

### 2.3 Search strategy and information sources

A comprehensive and systematic literature search was conducted between 7 and 12 December 2025 across multiple bibliographic and regional databases to identify all relevant studies on paediatric MASLD in Sub-Saharan Africa. The strategy combined controlled vocabulary (for indexed databases) with free-text keywords to maximize sensitivity and ensure inclusion of both contemporary MASLD terminology and legacy NAFLD literature. To mitigate the underrepresentation of African research in major global indices, targeted searches of African regional databases and grey literature sources were also performed. Search strings used include:

• PubMed/MEDLINE (MeSH-based and free-text search):

*(“Metabolic-associated steatotic liver disease”[TW] OR “MASLD”[TW] OR “Non-alcoholic fatty liver disease”[TW] OR “NAFLD”[TW])*.

*AND (“Paediatrics”[MeSH] OR “child”[TW] OR “adolescent”[TW])*.

*AND (“Africa”[MeSH] OR “Africa, South of the Sahara”[MeSH] OR “Sub-Saharan*.

*Africa”[TW])*.

• Embase (Exploded subject headings and text words):

*(‘metabolic associated steatotic liver disease’/exp OR ‘masld’ OR ‘nonalcoholic fatty liver’/exp*.

*OR ‘nafld’)*.

*AND (‘paediatrics’/exp OR ‘child’/exp OR ‘adolescent’/exp)*.

*AND (‘africa’/exp OR ‘sub-saharan africa’/exp)*.

• Cochrane Library (Title, Abstract, and Keyword fields):

*MASLD AND Paediatric AND Africa*.

• African Journals Online (AJOL):

*(MASLD OR NAFLD OR “fatty liver”)*.

*AND (children OR paediatric OR adolescent)*.

*AND (Africa)*.

To minimize publication and indexing bias, the search was supplemented with grey literature from WHO-AFRO reports and relevant African conference proceedings. To ensure a comprehensive conceptual framework, the search results were augmented by manual hand-searching of reference lists and the inclusion of foundational literature on genomics and metabolic pathophysiology.

### 2.4 Study selection and data synthesis

Study selection followed the PRISMA-ScR (Preferred Reporting Items for Systematic reviews and Meta-Analyses extension for Scoping Reviews) guidelines. Two reviewers (BB and PNO) independently screened titles and abstracts for relevance, followed by a full-text review of potentially eligible articles. Discrepancies between reviewers were resolved through consensus-based discussion. Data were extracted using a standardized charting tool capturing bibliographic details, diagnostic terminology, study population characteristics, and key findings. Given the heterogeneity in diagnostic methods (e.g., varying ALT cutoffs, ultrasound vs. elastography) and the scarcity of uniform datasets from SSA, a statistical meta-analysis was not feasible. Instead, a narrative synthesis was conducted. Findings were thematically organized to construct a comparative “two-speed” epidemiologic model, contrasting established global patterns with the emerging, unique clinical phenotype observed in Sub-Saharan Africa.

### 2.5 Evidence appraisal and quality assessment

A basic quality appraisal was performed to characterize the strength of the evidence base. Included studies were evaluated based on study design (longitudinal vs. cross-sectional), diagnostic gold-standard proximity (biopsy/MRI-PDFF vs. ultrasound/ALT), and population representation (population-based vs. clinic-based cohorts). The certainty of prevalence estimates in Sub-Saharan Africa (SSA) was low due to the predominance of small, clinic-based datasets which limits their generalizability.

## 3 Results

### 3.1 Search results and study characteristics

**Figure 1:**
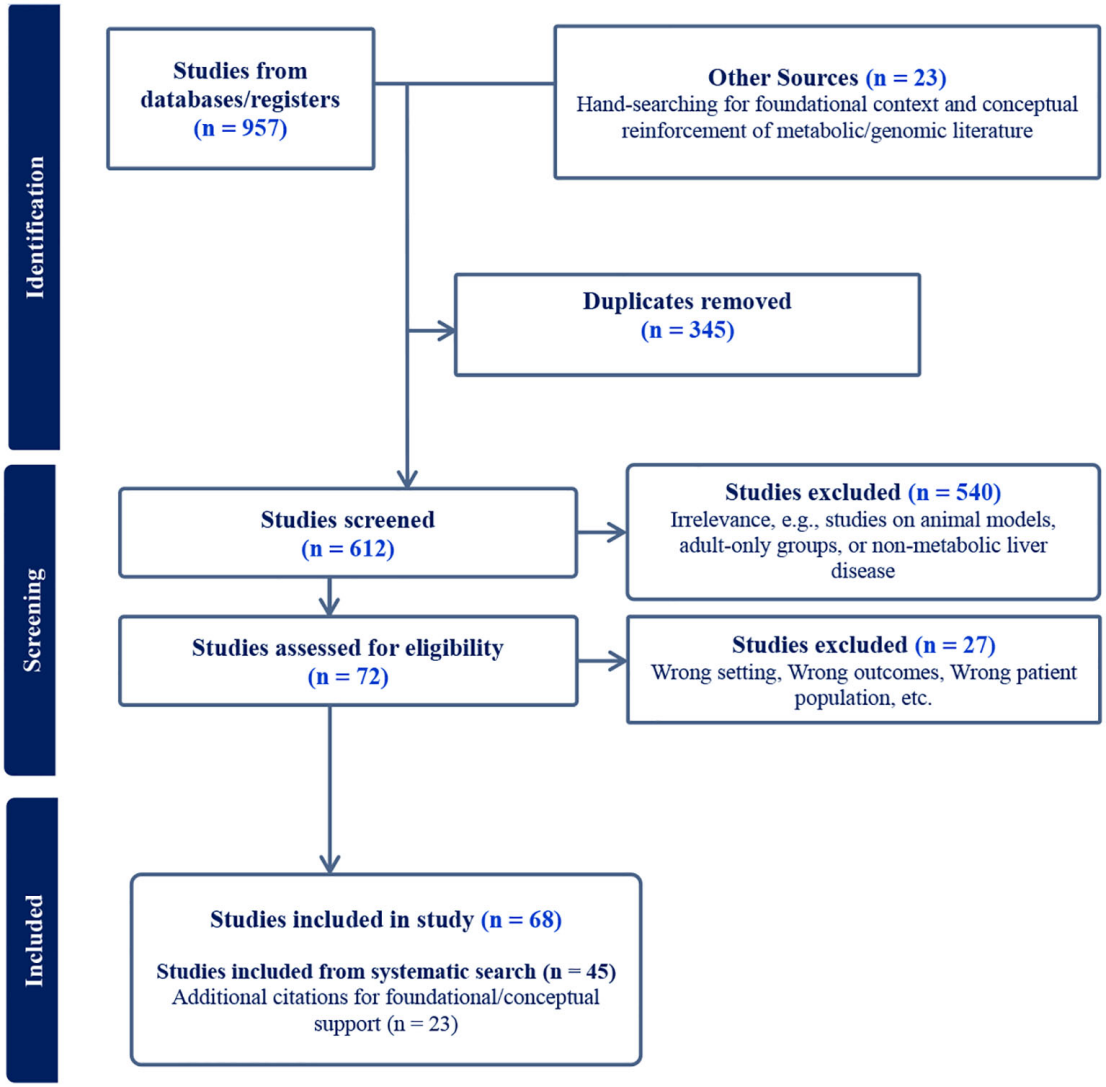
PRISMA-ScR flow diagram.

The initial database search yielded a total of 957 citations. After removal of duplicates, 612 unique records remained for title and abstract screening. Of these, 540 were excluded for irrelevance, including studies restricted to adult-only populations, animal models, or non-metabolic liver disease. A total of 72 full-text articles were assessed for eligibility. Following full-text review, 45 studies met the inclusion criteria. These included 40 global epidemiological studies and 5 studies involving primary data collection or systematic reviews conducted within the African region. An additional 23 citations were utilized to provide foundational context, analogical support for population-specific clinical traits, and conceptual reinforcement for the “African Paradox” discussion, bringing the total reference count to 68 (**Figure 1**). The African dataset comprised cohorts from Kenya, South Africa, and Egypt, alongside broader regional systematic reviews. Overall, the included studies relied predominantly on ultrasonography and liver enzyme measurements for diagnosis, with a marked scarcity of biopsy-confirmed or elastography-based data, particularly within the SSA context. A summary of the evidence certainty and primary methodological limitations is provided in **Box 1**.

Box 1 **Evidence quality and certainty appraisal**

**Purpose:** To formally characterize the strength and limitations of the evidence base informing MASLD epidemiology, diagnostics, and risk stratification, with particular emphasis on Sub-Saharan Africa.

Global prevalence (High-income countries and Asia):

*Moderate to high certainty*: This evidence is derived from large, population-representative longitudinal cohorts, registry-based surveillance, and multiple meta-analyses using standardized diagnostic criteria.

Sub-Saharan Africa prevalence:

*Low to very low certainty:* The available evidence is constrained by the scarcity of population-based studies, reliance on small clinic-derived cohorts from a limited number of urban centers (e.g., Nairobi and Cape Town), heterogeneous diagnostic definitions, and the absence of systematic surveillance systems.

Diagnostic tools in SSA:

*Low certainty:* Most SSA studies rely on non-gold standard modalities, particularly serum ALT and ultrasonography, which have limited sensitivity for mild steatosis and poor specificity for steatohepatitis and fibrosis. The near absence of biopsy-confirmed or MRI-PDFF data substantially limits the ability to quantify advanced disease or MASH prevalence.

Genetic and ancestry-related risk in SSA:

*Moderate certainty:* Evidence from ancestry-informed genomic studies supports population-level differences in MASLD susceptibility and progression, although region-specific and paediatric genomic data from SSA remain sparse.

### 3.2 Epidemiology

#### 3.2.1 global epidemiology of paediatric MASLD

The global burden of paediatric Metabolic-Associated Steatotic Liver Disease (MASLD) has increased dramatically over the past fifteen years, mirroring the trajectory of the childhood obesity pandemic. Evidence from 2010 to 2025 confirms that MASLD is no longer a rare clinical entity but a pervasive public health challenge affecting a significant proportion of children and adolescents worldwide (1, 2). However, prevalence estimates reported here are largely derived from NAFLD-era studies using ultrasonography or ALT proxies, which may not perfectly align with contemporary MASLD diagnostic requirements. In the general paediatric population, prevalence range between 7.6% and 14%, reflecting both methodological differences and rising trends over time (4, 6). Longitudinal data from the United States indicate that the prevalence of suspected fatty liver disease in adolescents has more than doubled over the past two decades, increasing from approximately 3.9% in the early 1990s to nearly 11% in later years (38, 39).

Prevalence is not uniform across demographic groups. A consistent male predominance of approximately 2:1 is observed, likely linked to sex hormone influences during puberty, with androgens promoting visceral fat deposition and hepatic lipid accumulation, while oestrogens appear protective (1, 26). Age-related prevalence peaks during late adolescence (15–19 years), corresponding to the physiological insulin resistance of puberty, which accelerates hepatic fat deposition when superimposed on excess adiposity (10).

Among high-risk clinical populations, particularly children with obesity and T2DM, MASLD prevalence is markedly higher. Children attending specialized obesity clinics show prevalence rates around 34.2%, while broader community-based reviews report approximately 41.2% of overweight or obese children affected (6). This emphasizes the strong pathophysiological link between excess adipose tissue and ectopic fat deposition in the liver (40). Dysglycaemia is also common within paediatric MASLD populations. In a U.S. multicentre cohort of 675 biopsy-confirmed cases, 23.4% had prediabetes and 6.5% had T2DM, with both conditions significantly increasing the likelihood of steatohepatitis (41), highlighting the bi-directional link between hepatic steatosis and impaired glucose regulation. A meta-analysis of paediatric T2DM cohorts (n= 4,510) revealed a pooled global prevalence of approximately 33.8% (95% CI 24.2–44.1%). Studies using combined liver-function tests and ultrasonography reported rates of about 49%, while MRI-based assessments identified MASLD in 54–55% (42), emphasizing the universality of this metabolic association. Emerging data also indicate elevated MASLD prevalence among children with Type 1 Diabetes (T1DM), particularly those with poor glycaemic control; a study in Egypt reported 62.2% prevalence (43), challenging the notion that MASLD is exclusively a T2DM-associated disorder.

Geographically, North America reports the highest prevalence in overweight children (43.6%), while Asia has rapidly converged with similar prevalence rates (42.1%) due to earlier metabolic derangements at lower BMI thresholds (6). The Middle East and North Africa have experienced similar increases, driven by environmental and lifestyle factors, with countries such as Egypt, Kuwait, and Qatar exhibiting some of the highest adolescent MASLD prevalence globally (21).

#### 3.2.2 Epidemiology of paediatric MASLD in sub-Saharan Africa

The evidence for paediatric MASLD in SSA remains sparse but growing and suggests that it occurs in African children especially among those with overweight or obesity (**Table 1**). Notably, while prevalence in Sub-Saharan African cohorts such as those in Kenya (26.2%) and South Africa (9%), is substantial, it remains markedly lower than the 62.2% burden reported in North Africa among children with Type 1 Diabetes in Egypt. For example, a recent cross−sectional study in Nairobi, Kenya reported a prevalence of 26.2% among 103 overweight or obese children aged 6–18 years using liver ultrasonography. This study also found that obesity (versus overweight) conferred a four−fold higher odds of NAFLD, and that older children (13–18 years) had significantly higher prevalence than younger ones (44). A recent global meta-analysis of MAFLD in children and adolescents with overweight or obesity included data from Africa and reported a pooled prevalence estimate of 31.1% (95% CI 23.8–38.5%) for the African subgroup; the authors noted that this estimate was primarily driven by the Nairobi study, highlighting both the scarcity of data and the need for further regional research (6). In contrast, data from South Africa highlight a different risk profile. A recent study reported a MASLD prevalence of 9% among children with perinatally acquired HIV compared with 1% in uninfected controls. Notably, significant hepatic steatosis was observed even in children classified as lean (BMI z-score ≤ 1), emphasizing the role of non-obesity-related metabolic drivers in this setting (45).

**Table 1:** Characteristics of selected studies on paediatric MASLD in Africa (2010–2025).

Author/Year	Country (Region)	Population (N)	Age (Yrs)	Diagnostic method (Definition Used)	Prevalence/Key findings
Sub-Saharan Africa					
Mburu et al. (2023) (44)	Kenya (East)	N=103 (Overweight/Obese)	6–18	Ultrasonography (NAFLD)	26.2% prevalence in overweight/obese children. Obesity conferred a 4-fold higher risk than overweight alone.
Rose et al. (2022) (45)	South Africa (South)	N=215 (Perinatal HIV & Controls)	12–15	Transient Elastography (FibroScan CAP) (NAFLD)	9% in HIV-positive children vs. 1% in controls. Crucial Finding: Steatosis was significantly more common in lean (BMI z-score ≤ 1) HIV-positive children, supporting the “lean MASLD” phenotype in SSA.
Sindato et al. (2025) (7)	Pooled SSA	Systematic Review (Mixed Ages)	Mixed	Various (MAFLD/MASLD)	29.2% overall prevalence in SSA. Regional breakdown: West (34.4%), South (26.9%), East (24.6%).
North Africa					
Abdallah et al. (2023) (43)	Egypt (North)	N=74 (Type 1 Diabetes)	8–18	Ultrasonography & Elastography (NAFLD)	62.2% prevalence in T1DM children. 26% had significant fibrosis (F2–F4). Highlights the high burden in T1DM populations in Africa.
Africa					
Jia et al. (2025) (6)	Pooled Africa	Meta-analysis (Multiple studies)	2–18	Various (MAFLD)	31.1% pooled prevalence among children with overweight/obesity in the African region.

At the same time, broader narrative and review−level literature in Africa continues to emphasize how severely under−studied NAFLD/MASLD remains on the continent. A review argued that extant prevalence estimates, often from small, opportunistic, or adult studies, likely underestimate the true burden, particularly in children, because of underdiagnosis, limited screening capacity, and low clinical suspicion (46).

### 3.3 Risk Factors and pathophysiology

#### 3.3.1 Environmental and metabolic drivers

Paediatric MASLD develops through a complex interplay of environmental and metabolic factors. A comparison of the distinct risk factor profiles between the Western model and the Sub-Saharan African context is presented in **Table 2**. Expansion of visceral adipose tissue is particularly critical, as it increases the flux of free fatty acids to the liver, overwhelming hepatic lipid oxidation and secretion (40). Evidence from Kenyan cohorts demonstrates a four-fold higher risk of fatty liver in obese children compared to overweight peers, illustrating the dose-dependent relationship between adiposity and hepatic steatosis (7, 44).

**Table 2:** Comparative Risk Factor Profile: Western vs. Sub-Saharan African Paediatric MASLD.

Risk Domain	Western Model (HIC)	Sub-Saharan African Model (SSA)
Primary Driver	Caloric Excess (Obesity)	“Double Burden”: Malnutrition (Stunting), and Rapid Nutritional Transition
Dietary Factors	Ultra-processed foods, Saturated Fats	High-Fructose (Liquid Sugar), Carbohydrate mono-diets, and potential environmental co-factors such as mycotoxin exposure (Aflatoxin)
Genetic Susceptibility	High prevalence of PNPLA3 (I148M) risk allele (especially Hispanic/Caucasian)	“African Paradox”: Low frequency of PNPLA3 risk allele (~13%), yet high insulin resistance
Environmental Exposures	Endocrine Disrupting Chemicals (EDCs)	EDCs, Antiretroviral Therapy (ART) exposure (lifelong), and Chronic Inflammation (HIV/TB/Malaria)
Clinical Phenotype	High Liver Fat + High Visceral Fat	Lower Liver Fat (Steatosis), High Insulin Resistance, and “Lean” MASLD

Dietary factors, particularly high-fructose intake from sugar-sweetened beverages, contribute significantly to disease pathogenesis. Fructose metabolism bypasses phosphofructokinase regulation, promoting unregulated *de novo* lipogenesis while depleting hepatic ATP and generating uric acid, which induces oxidative stress (12, 47). Sedentary behaviours, exacerbated by urbanization and academic screen-based activities, further compounds insulin resistance by reducing skeletal muscle glucose uptake and fatty acid oxidation (48).

Endocrine-disrupting chemicals, including bisphenol A, phthalates, and perfluoroalkyl substances, are ubiquitous in urban African environments and emerging evidence suggest that they act as obesogens, disrupting hepatic lipid metabolism and insulin signalling, lowering the threshold for MASLD development even in moderately overweight children (11). Maternal obesity and gestational diabetes also create an intrauterine environment of fuel excess, predisposing offspring to early-onset steatosis (13).

#### 3.3.2 Genetic factors and ethnic differences in MASLD risk

Genetic predisposition is a critical determinant of MASLD susceptibility. The PNPLA3 I148M (rs738409) variant is the strongest known genetic risk factor (14). Recent data from a large multi-ethnic biobank demonstrate that the “G” risk allele is significantly more common in Hispanic/Latino populations (47.2%), less frequent in European-ancestry individuals (22.8%), and least frequent in African-American individuals (13.7%) (49). In addition, an admixed-population study revealed that among individuals with MASLD, higher African genetic ancestry was associated with a lower frequency of the PNPLA3 G allele suggesting a possible ancestry-associated protective factor against steatosis (25, 27). These findings suggest that while lower PNPLA3 risk-allele frequency may partly explain the “African paradox” of reduced hepatic fat in African-ancestry populations, genetic variation is only a modifying factor, and environmental, metabolic, and lifestyle factors remain key determinants of MASLD risk. Moreover, reduced hepatic fat does not equate to reduced metabolic risk, as insulin resistance and cardiovascular complications remain prevalent (50).

The TM6SF2 rs58542926 (E167K) variant, a loss-of-function allele, impairs hepatic VLDL secretion, thereby promoting intrahepatic fat accumulation while concurrently lowering circulating lipids such as LDL and triglycerides. This dual effect may obscure cardiovascular risk when using standard lipid-based risk profiling, despite increased hepatic fat burden in carriers (51, 52). Polygenic risk scores incorporating PNPLA3, TM6SF2, and HSD17B13 show predictive potential in Western cohorts but remain largely unvalidated in African populations (28).

Furthermore, the observation of population-specific clinical traits, such as the African lean phenotype, parallels findings in other genetic disorders in which distinct genotyping profiles, driven by high variability in causative genes such as COL1A1 and COL1A2, which encode the collagen type I alpha chains, give rise to divergent phenotypic expressions across populations with osteogenesis imperfecta (53–55). Beyond PNPLA3, emerging evidence suggests that the WFS1 gene may be central to the “African Paradox” observed in SSA. This WFS1 gene is a critical regulator of the endocrine system and endoplasmic reticulum (ER) stress. Its variants have been linked to pronounced insulin resistance and diabetes susceptibility while potentially conferring resistance to high-fat diet induced hepatic steatosis (56, 57). Given that WFS1 profiles exhibit significant inter-population variability, this gene represents a plausible mechanistic candidate for the dissociation between systemic metabolic dysfunction and visible liver fat in African-ancestry children (58, 59).

### 3.4 Screening and diagnostic approaches

#### 3.4.1 Guideline recommendations and practical feasibility in SSA

Major paediatric hepatology societies recommend screening children with obesity starting at 9–11 years using serum ALT and ultrasonography (9, 33). However, reliance on ALT alone is problematic. Reference ranges often remain too high, leading to under-detection of early disease, and ALT levels correlate poorly with histological severity (48). Ultrasound, although widely available, has limited sensitivity for mild steatosis (<33% hepatic fat) and is operator-dependent (60). Advanced modalities, such as vibration-controlled transient elastography (FibroScan) and MRI-PDFF, offer superior diagnostic accuracy, including quantification of liver fat and fibrosis staging (35). However, in SSA these tools are scarcely available in tertiary centres, are cost-prohibitive, and inaccessible for routine screening (34, 36). Liver biopsy remains the gold standard for diagnosing steatohepatitis and fibrosis even in children and adolescence (4), but is rarely feasible due to procedural risk and shortage of paediatric hepatology expertise in the SSA region.

#### 3.4.2 Practical innovations for screening in resource-limited settings

Recent paediatric studies have explored simplified screening algorithms suitable for low-resource settings. In obese and overweight children and adolescents, waist-to-height ratio (WHtR), an inexpensive and easy-to-measure anthropometric index, has emerged repeatedly as a superior predictor of hepatic steatosis compared with BMI z-score or waist−hip ratio (61, 62). Although optimal WHtR cutoffs vary by study (e.g., about 0.47–0.48 in some) and likely depend on population anthropometry and demographic factors, this approach offers a practical first step in community-level screening.

Regarding fibrosis risk stratification, non-invasive scores such as the AST-to-platelet ratio index (APRI) have shown modest utility in Paediatric NAFLD, with limited sensitivity and specificity, whereas other adult-derived scores (FIB-4, NFS) generally perform poorly in children (63–65). These tools may have potential use as preliminary “rule-out” triage instruments in settings lacking elastography or biopsy facilities, but they cannot reliably replace histology or elastography for diagnosing significant fibrosis. Further validation is needed, especially in diverse and resource-constrained settings.

## 4 Discussion

### 4.1 Summary of the key findings

This scoping review synthesizes a rapidly evolving body of evidence revealing a “two-speed” epidemic of paediatric MASLD which is characterized by established high prevalence in the West and a rapidly accelerating, yet under-diagnosed, burden in SSA. Available data confirms that MASLD has transitioned from a rare clinical entity to a ubiquitous public health challenge that mirrors the global obesity pandemic, with a distinct predilection for male adolescents and a peak incidence coinciding with the physiological insulin resistance of puberty. Crucially, the evidence refutes the historical assumption that African children are spared as prevalence rates in high-risk groups within the region now rival global averages. This is driven by a combination of rapid urbanization, dietary transitions toward ultra-processed foods, and the unique “double burden” of coexisting malnutrition and obesity. However, this escalating crisis remains largely invisible to health systems due to a critical disconnect between the complex, multifactorial drivers of the disease, ranging from environmental obesogens to the “African Paradox” of genetic susceptibility, and a continued reliance on diagnostic tools that may under-detect the specific phenotypic presentation of African paediatric populations.

### 4.2 Interpretation and contextualization of the findings

The observed differences in MASLD prevalence between high-income regions and SSA reflect a complex interplay of lifestyle transitions and diagnostic capacity rather than purely biological differences (21, 46). Globally, the rising prevalence aligns with parallel increases in childhood obesity, sedentary behaviours, and exposure to energy-dense diets, confirming the central role of environmental factors in MASLD pathogenesis (1, 2). In SSA, existing prevalence estimates appear lower very likely as a result of underdiagnosis, limited screening infrastructure, and the reliance on less sensitive modalities like ALT or basic ultrasound, rather than a true absence of disease (45, 46, 66). Urban SSA populations, where obesogenic diets and sedentary lifestyles are emerging, show prevalence rates approaching those in high-income countries (7, 44), suggesting that lifestyle factors can rapidly override any population-level genetic protection.

Also, the concept of an “African Paradox” is supported by genetic data, particularly the lower prevalence of PNPLA3 and TM6SF2 risk alleles in populations of African ancestry (25, 27, 52). Yet, the paradox does not translate to metabolic protection. African children frequently display insulin resistance, dyslipidaemia, and early markers of cardiovascular risk (40, 50). This dissociation between liver fat and systemic metabolic risk highlights the limitations of conventional diagnostic strategies focused solely on hepatic imaging. Furthermore, it is critical to differentiate between diaspora cohorts and children living in SSA, as local environmental stressors may yield different phenotypic expressions. It suggests that, in African populations, metabolic risk may precede overt hepatic steatosis, requiring clinicians and public health practitioners to adopt a broader risk assessment approach that integrates anthropometric, biochemical, and environmental indicators.

Crucially, the rise in MASLD prevalence in SSA emphasizes the key role of environmental drivers. High-fructose diets, rapid urbanization, reduced physical activity, and exposure to endocrine-disrupting chemicals collectively create a metabolic milieu conducive to liver fat accumulation (11, 12, 48). This “environmental overload” may mask genetic protective effects, demonstrating the dynamic interaction between genes and environment. Additionally, early-life nutritional deficits, particularly stunting and low birth weight, may sensitize children to later metabolic derangements via thrifty phenotype programming (13, 22), meaning that even modest exposure to excess calories or obesogens can disproportionately impact hepatic metabolism.

It is important to note that MASLD is not simply a liver-specific disease. This is because even in the absence of severe steatosis, African children with overweight or obesity exhibit insulin resistance, dyslipidaemia, and increased cardiometabolic risk (19, 40). This suggests that MASLD is not merely a liver-specific disease but a hepatic manifestation of a broader metabolic dysfunction, particularly in populations with lower genetic susceptibility to fat accumulation. Recognizing this helps contextualize the SSA burden as standard steatosis-centric screening approaches may underestimate the true metabolic risk. This emphasizes the need for holistic risk stratification strategies.

By and large, the interplay of prevalence, genetics, and environment has direct implications for screening in SSA. The lack of sensitive imaging modalities and validated non-invasive scores for children means many at-risk children remain undetected until metabolic complications emerge (30, 34). This gap illustrates that the scarcely available prevalence data in SSA likely underestimate true disease burden, and innovations such as WHtR and APRI offer critical, locally appropriate tools for early detection. From a public health perspective, this reinforces the need to integrate simple anthropometric and laboratory-based metrics into routine paediatric assessments in resource-limited settings.

### 4.3 Implications for clinical practice, public health and healthcare systems in SSA

Clinically, the current reliance on standard diagnostic markers creates a risk of misclassification in SSA. Serum ALT, the most widely available screening tool, frequently utilizes reference ranges derived from adult or Western populations that are set too high for African children, leading to the under-detection of early hepatic injury (9, 48). Furthermore, standard ultrasonography is insensitive to the mild-to-moderate steatosis often seen in the African phenotype and is highly operator-dependent, further compounding diagnostic gaps (35, 60).

To mitigate these challenges, the adoption of lower, biologically appropriate ALT thresholds, specifically >22 U/L for girls and >26 U/L for boys, is critical (9). Although these lower ALT thresholds improve sensitivity and are recommended by NASPGHAN, their use in SSA should be contextual, as these cutoffs were derived largely from non-African populations and may increase false positives in regions where endemic infections such as viral hepatitis, schistosomiasis, or malaria can cause mild transaminase elevations. Regionally validating and implementing these tighter cutoffs in clinical practice will ensure that at-risk children are not missed due to inappropriate laboratory norms, allowing for earlier intervention before fibrosis progression occurs.

From a public health perspective, the scarcity of advanced imaging modalities like elastography necessitates the adoption of “frugal innovations” for mass screening. **Table 3** outlines proposed diagnostic adaptations, including biologically appropriate ALT cutoffs and anthropometric markers, suitable for resource-limited settings. The Waist-to-Height ratio (WHtR) emerges as a superior alternative to BMI, offering a simple, zero-cost metric that correlates strongly with visceral adiposity, the primary driver of metabolic risk in this population (61, 62). A validated cutoff of ≥0.48 provides a practical triage tool that can be easily implemented in school health programs, vaccination clinics, or community nutritional surveys. This proposed cutoff should be regarded as provisional, as regional differences in growth patterns and stunting may modify its relationship with visceral adiposity, necessitating local validation. Unlike complex risk scores requiring laboratory inputs, this anthropometric approach allows for the equitable identification of children at risk for MASLD and metabolic syndrome even in the most resource-constrained rural settings.

**Table 3:** Comparison of diagnostic modalities for MASLD in resource-limited settings.

Diagnostic tool	Standard (Western) criteria	Limitations in SSA context	Proposed “Frugal” adaptation for SSA
Serum ALT	Cutoff often >45 U/L (Adult norms)	Misses early inflammation; poorly correlated with histology in African children.	Adopt Biologically Appropriate Cutoffs: Boys >26 U/L Girls >22 U/L
Ultrasonography	First-line screening	Low sensitivity for mild steatosis (<33% fat); highly operator-dependent; scarcity of radiologists.	Use primarily to rule out other pathology; do not rely on it to exclude MASLD in high-risk lean children.
Anthropometry	BMI Z-Score (Obesity focused)	Fails to detect central adiposity in “stunted-overweight” or “lean-metabolic” children.	Waist-to-Height Ratio (WHtR): Cutoff ≥ 0.48. Superior predictor of metabolic risk and visceral fat in African populations.
Advanced Imaging	MRI-PDFF/FibroScan	Prohibitively expensive; unavailable in most district hospitals.	Task-Shifting: Use APRI Score (AST-to-Platelet Ratio) as a preliminary “rule-out” tool for fibrosis where FibroScan is absent.

At the health-system level, the severe shortage of paediatric hepatologists and limited tertiary infrastructure render the specialized Western model of care unsustainable for SSA. Addressing this gap requires a paradigm shift toward task-shifting models, wherein nurses and clinical officers, who already shoulder the burden of HIV and TB care (30, 31), are trained to screen for metabolic liver disease using these simplified algorithms. Integrating MASLD screening into existing chronic care infrastructures, such as paediatric diabetes and HIV clinics, offers a scalable solution that leverages existing workforce capacity. This integrated approach not only maximizes resource utilization but also ensures that metabolic liver health is treated as a core component of non-communicable disease management rather than a separate sub-specialty. Although EDCs and ART effects represent significant emerging evidence in the SSA context, they are currently viewed as regional modifiers that operate alongside the core established drivers of adiposity and nutritional transition.

**Figure 2:**
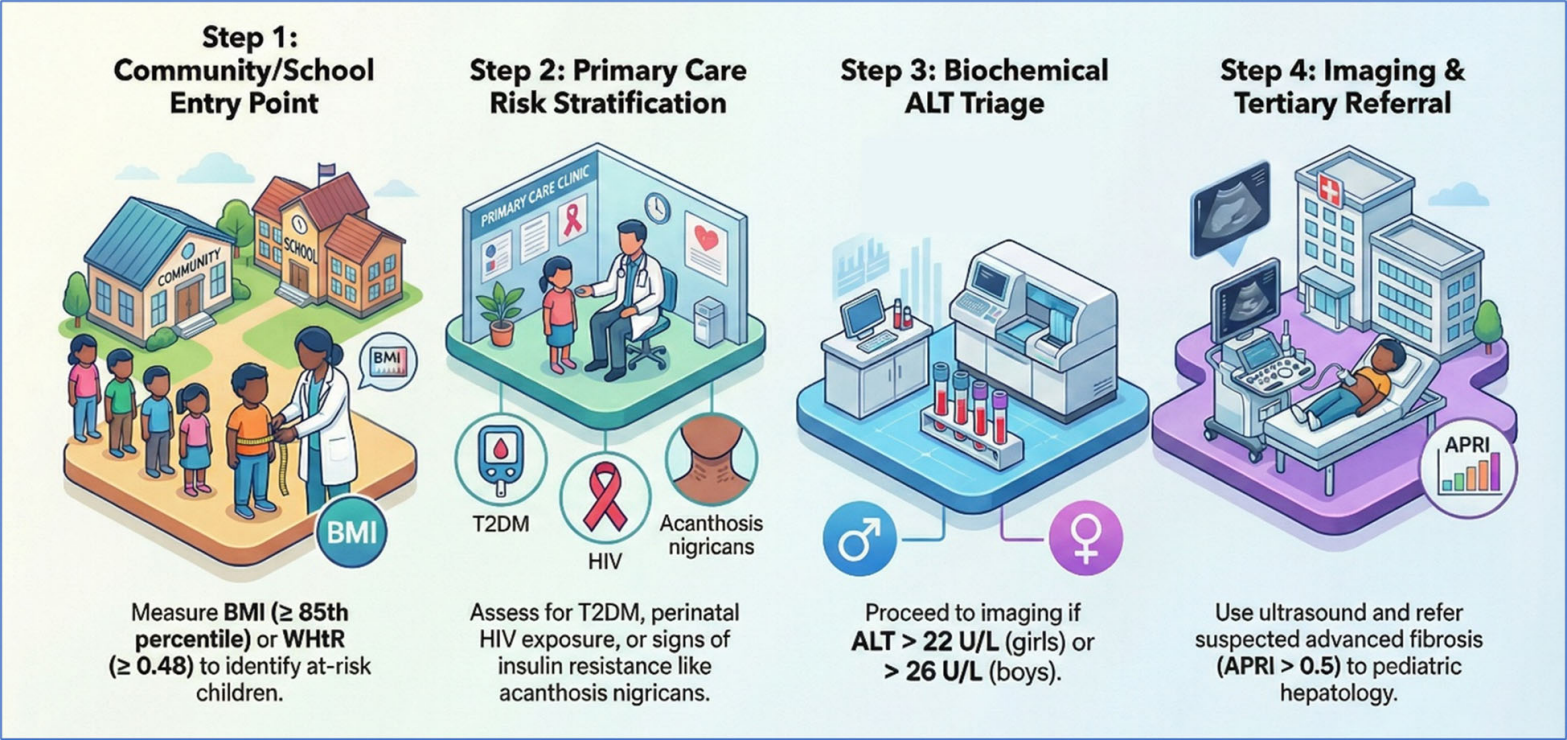
Proposed SSA-Adapted Screening Pathway for Paediatric MASLD. This model integrates community-level anthropometry (WHtR) with clinical risk factors (HIV/T2DM) and refined biochemical thresholds to facilitate early detection in resource-limited settings.

To guide this transition, **Figure 2** presents a proposed clinical decision pathway adapted for the SSA context, prioritizing low-cost anthropometric triage and biologically appropriate biochemical screening.

### 4.4 Strengths and limitations of the study

This review is strengthened by a comprehensive search strategy that bridged global databases with regional sources, including African Journals Online (AJOL) and grey literature, ensuring that African perspectives were adequately represented. Additionally, the inclusion of recent data (2020–2025) allows for a timely synthesis of the new MASLD nomenclature and provides a unique integration of genetic insights (PNPLA3) specifically within the African context, moving the discourse beyond simple prevalence to a more comprehensive mechanistic understanding.

However, the interpretation of these findings is limited by significant heterogeneity in diagnostic methods, ranging from varying ALT assays and operator-dependent ultrasound quality to rare MRI usage, which makes the potential conduct of a formal meta-analysis problematic. Also, the ability to define broad regional patterns is limited by reliance on sparse data, derived primarily from a small number of isolated clinical cohorts rather than large-scale population-based surveillance (**Table 1**). Another critical gap remains the absence of biopsy-based studies in SSA without which precise estimates of steatohepatitis (MASH) prevalence are unavailable, meaning current figures for progressive disease remain approximations. Furthermore, existing data is heavily skewed toward urban centres, potentially masking the burden in rural populations, and currently available polygenic risk scores remain unvalidated for African genome. Nonetheless, this synthesis provides a detailed, contemporary overview of paediatric MASLD, emphasizing the urgent need for locally-adaptable screening and intervention strategies in Africa.

### 4.5 Research gaps and future directions

To bridge the gap between global precision medicine and the realities of the SSA context, future research must address the following critical deficits:

- Available African data is cross-sectional, preventing definitive conclusions about disease progression. Future cohorts must track the specific trajectory from childhood stunting or obesity to adult MASLD and cirrhosis to rigorously test the “thrifty phenotype” hypothesis (3, 7).
- Current genetic risk scores are of Caucasian origin and remain untested in African children. Large-scale, region-specific Genome-Wide Association Studies (GWAS) are required to identify unique variants that may explain the “lean” MASLD phenotype and inform locally relevant risk stratification (14, 28).
- Non-invasive fibrosis scores lack rigorous validation against liver biopsy in African paediatric populations. Future research must prioritize biopsy-proven correlations to accurately quantify the burden of steatohepatitis (MASH) and prevent false reassurance or missed diagnoses of advanced fibrosis (34, 64).
- There is a need to evaluate lifestyle interventions targeting fructose reduction and EDC avoidance, alongside cost-effectiveness studies of implementing anthropometric screening (WHtR) in schools to guide national health policy (11, 12).
- Future research should leverage transcriptomic analysis of resident SSA patients to identify specific metabolic pathways or repetitive genetic elements that are uniquely altered in the African metabolic milieu (67, 68).
- To move beyond cross-sectional associations, Mendelian randomization should be employed to rigorously evaluate the causal interactions between environmental drivers, perinatal programming, and the “lean” MASLD phenotype in SSA populations (14, 28).

Generally, large-scale, population-based studies in diverse SSA settings are needed to properly define prevalence, risk-factor architecture, and socio-demographic stratifiers so that these policies can be appropriately sized and targeted.

## 5 Conclusion

This scoping review synthesizes a rapidly evolving body of evidence positioning paediatric MASLD not merely as a consequence of caloric excess, but as a complex, multifactorial pathology driven by the collision of genetic susceptibility, environmental toxins, and the “double burden” of malnutrition. Although the global narrative has shifted toward advanced phenotyping and precision medicine, the implications for Sub-Saharan Africa are distinct and urgent. The region faces a “two-speed” epidemic where rapid urbanization and emerging risk factors like endocrine-disrupting chemicals are outpacing health system adaptation. In a context where the “African Paradox” may result in the under-detection of metabolic risk using standard imaging criteria due to the lower prevalence of the PNPLA3 risk allele, the continued reliance on expensive, Western-calibrated diagnostics creates a significant risk of misclassification, potentially delaying early intervention. Consequently, shifting the paradigm from high-cost imaging to resource-appropriate innovation, specifically utilizing task-shifting and validating scalable anthropometric markers like Waist-to-Height Ratio, represents a high-priority public health frontier, offering a sustainable strategy to avert a future wave of end-stage liver disease in the developing world.

## Data Availability

The original contributions presented in the study are included in the article/supplementary material. Further inquiries can be directed to the corresponding author.

## Glossary

AJOL: African Journals Online
ALT: Alanine Aminotransferase
APRI: AST-to-Platelet Ratio Index
ART: Antiretroviral Therapy
AST: Aspartate Aminotransferase
ATP: Adenosine Triphosphate
BMI: Body Mass Index
CVD: Cardiovascular Disease
EDCs: Endocrine-Disrupting Chemicals
FIB-4: Fibrosis-4 Index
GWAS: Genome-Wide Association Studies
HICs: High-Income Countries
HIV: Human Immunodeficiency Virus
JBI: Joanna Briggs Institute
LMICs: Low- and Middle-Income Countries
MAFLD: Metabolic Dysfunction-Associated Fatty Liver Disease
MASH: Metabolic-Associated Steatohepatitis
MASLD: Metabolic-Associated Steatotic Liver Disease
MeSH: Medical Subject Headings
MRI-PDFF: Magnetic Resonance Imaging Proton Density Fat Fraction
NAFLD: Non-Alcoholic Fatty Liver Disease
NCDs: Non-Communicable Diseases
NFS: NAFLD Fibrosis Score
PCC: Population, Concept, and Context
SSA: Sub-Saharan Africa
T1DM: Type 1 Diabetes Mellitus
T2DM: Type 2 Diabetes Mellitus
TB: Tuberculosis
VLDL: Very Low-Density Lipoprotein
WHO: World Health Organization
WHtR: Waist-to-Height Ratio.
